# Enhancing governance and health system accountability for people centered healthcare: an exploratory study of community scorecards in Afghanistan

**DOI:** 10.1186/s12913-015-0946-5

**Published:** 2015-07-31

**Authors:** Anbrasi Edward, Kojo Osei-Bonsu, Casey Branchini, Temor shah Yarghal, Said Habib Arwal, Ahmad Jan Naeem

**Affiliations:** Department of International Health, Johns Hopkins Bloomberg School of Public Health, 615 N Wolfe St, Baltimore, MD 21205 USA; Babson College, Mailbox #363, Babson Park, MA 02457 USA; Future Health Systems Consultant, CBHC Department, Ministry of Public Health, Kabul, Afghanistan; CBHC Department, Ministry of Public Health, Kabul, Afghanistan; Ministry of Public Health, Kabul, Afghanistan

**Keywords:** Community scorecards, Patient-centered healthcare, Afghanistan, Conflict/Post-Conflict

## Abstract

**Background:**

The premise of patient-centered care is to empower patients to become active participants in their own care and receive health services focused on their individual needs and preferences. Afghanistan has evidenced enormous gains in coverage and utilization, but the quality of care remains suboptimal, as evidenced in the balanced scorecard (BSC) performance assessments. In the United States and throughout Africa and Asia, community scorecards (CSC) have proved effective in improving accountability and responsiveness of services. This study represents the first attempt to assess CSC feasibility in a fragile context (Afghanistan) through joint engagement of service providers and community members in the design of patient-centered services with the objective of assessing impact on service delivery and perceived quality of care.

**Methods:**

Six primary healthcare facilities were randomly selected in three provinces (Bamyan, Takhar and Nangarhar) and communities in their catchment area were selected for the study. Employing a multi-stakeholder strategy, community members and leaders, health councils, facility providers, NGO managers, and provincial directorates were engaged in a five-phase process to jointly identify structural and service delivery indicators (about 20), score performance and subsequently develop action plans for instituting improvements through participatory research methods. Three rounds of CSC assessments were conducted in each community. Over 470 community members, 34 health providers, and other provincial ministry staff participated in the performance audits.

**Results:**

Structural capacity indicators including the number and cadre of service providers, particularly female providers, water and power supply, waiting rooms, essential medicines, and equipment scored low in the first round (30–50 %). Provider courtesy and quality of care received high scores (>90 %) throughout the study. Unrealistic community demands for ambulances and specialist doctors were mitigated by community education of entitlements described in the national standards for essential package of services. The joint interface meeting facilitated transparent dialogue between the community and providers and resulted in creative and participatory problem solving mechanisms and mobilization of resources.

**Conclusion:**

These results indicate the potential of the CSC as a tool for enhancing social accountability for patient-centered care. However, the process requires skilled facilitators to effectively engage communities and healthcare providers and adaptation to specific healthcare contexts.

**Electronic supplementary material:**

The online version of this article (doi:10.1186/s12913-015-0946-5) contains supplementary material, which is available to authorized users.

## Background

Afghanistan, like other fragile states, has yet to achieve a single Millennium Development Goal [1]. In the past decade, accelerated investments for revamping the health service delivery architecture have resulted in improvements in most service delivery performance domains, as illustrated by the balanced scorecard (BSC) [2] and subsequently declining trends in maternal and child mortality despite the methodological limitations in the estimates [3]. Sustaining these gains in the future is likely to be a challenge, as withdrawal of military forces may result in the potential exodus of developmental organizations and worsen the chronic workforce deficits.

The Afghanistan Basic Package of Health Services (BPHS) was designed based on the national policy and mission for equitable and quality care to the rural poor [4]. The BSC proved to be a successful management strategy for policy makers to provide oversight to contracted services, but results were seldom communicated to the frontline providers in the health facilities. Patient perspectives of services and engagement of community councils was integrated in the BSC domains, but the information was not optimized to ensure provider or community engagement for performance improvement.

Since 2005, the Community Based Healthcare Department (CBHC), at the Afghanistan Ministry of Public Health (MOPH) has instituted several community engagement strategies to ensure access to health services, including the deployment of over 29,000 community health workers, institution of community health councils and community health supervisors through the NGO contracting mechanisms. Some of these community strategies like the Partnership Defined Quality, Family Health Action Group, and the National Solidarity Program, which established over 30,000 community development councils reaching a population of over 10.5 million [5], attempt to integrate problem solving measures. Though these capacity-building and empowerment strategies have been successful in some contexts, facilitating receipt of funding for community projects, improving accountability and minimizing corruption through social audits and trust management, and wide variations in the execution of these strategies, may pose an additional burden for community health workers (CHW) oversight, as they are already overwhelmed with high task expectations for community healthcare.

Community capacity building and empowerment initiatives engaging communities in program planning, management, and financial oversight have illustrated some evidence of improved health outcomes, but replication at the national level remains a challenge, as the operating environment differs across settings [6–9]. Despite the evidence on the effectiveness of community engagement, it has become increasingly apparent that these outcomes can only be sustained in a framework of community support mechanisms, responsibility and management accountability [10, 11]. In conflict and transitional contexts, additional efforts are required to build trust in public services and reinforce community institutions, rather than undermining them.

Adapted from the concept of BSC for strategic management [12], the Community Scorecard (CSC), is a monitoring tool that is used for local monitoring and performance evaluation of services, projects, and even government administrative units by the communities themselves. It is a hybrid of the social audit and citizen report card with the aim of empowering communities and enhancing social responsiveness in the delivery of care and the opportunities to enhance progress toward outcomes. The CSC focuses on the community as the unit of analysis and solicits perceptions on quality, efficiency and transparency and tracks inputs, such as essential medicines, expenditures, monitors quality of services/projects, and generates a direct feedback mechanism between service providers and users, building local capacity and strengthening citizen voice and community empowerment. As the decision-making dynamics in communities vary, the process needs to be adapted to the local healthcare context.

In the United States, the CSC has been piloted as a strategic planning and performance management tool for communities to make informed decisions about critical issues for civic accountability [13, 14]. In Africa and Asia, the CSC has been successfully integrated with healthcare, education, sanitation and other sectors, resulting in increased community ownership to services, trust of public institutions and healthcare providers, and enhanced performance of health facilities, resulting in improved equity, access, quality and healthcare [15–17]. A randomized study on CSC in Uganda, illustrated reductions in child mortality, increased service utilization and improved perceived quality of care [18]. Pilot experiments by the Ministry of Planning in Vietnam and UNICEF and the Northern Ghana Network in Ghana have also demonstrated positive results as a tool for assessing social performance [19, 16].

In fragile states, with protracted conflicts, where trust of public institutions is low, CSC experiments have not been documented. This study was designed to determine the feasibility of executing CSC in Afghan communities, to complement the ongoing national BSC performance to determine if health service delivery can be improved with active community engagement.

## Methods

The exploratory study was conducted in 2012, in partnership with the CBHC Department, MOPH, and integrated into an ongoing national health services performance assessment. Following an evaluation of community engagement strategies at the national-level, the CBHC Department and other donors invested in community healthcare participated in a national workshop to illustrate the CSC methodology and results achieved in other countries. Initially two of the largest NGOs in Bamyan (Central) and Takhar (Northeast) provinces offered to participate in the study. In each province, two primary health facilities, either Basic Heath Centers (15,000–30,000 catchment area population) or Comprehensive Health Centers (30,000–60,000 catchment area population) were randomly selected from those already participating in the ongoing national assessment. These included facilities in Siadara and the Sarqol Valley of Yakawlang District in Bamyan, and Kalafgan and Namak Ab Districts in Takhar. Subsequently, two additional facilities; Bakhtan and Kuz Kunar in Nangarhar province were also included to ensure ethnic diversity. The study was concluded in September 2013.

Community assessment teams were recruited to participate in a ten-day training on BPHS standards, participatory research methods, the conduct of field surveys, and the CSC methodology, as adapted from the *CARE Malawi Manual* [20]. The teams comprised of male and female members who had prior experience in conducting quantitative and qualitative community based surveys and were familiar with participatory methods. A majority had engaged in several evaluations conducted nationally, and through the CBHC department, MOPH. Four-member teams (two males, two females) were deployed to each field site. The survey teams also received ongoing facilitation and follow-up during each round of data collection to ensure quality and safety precautions. Focus group discussion (FGD) guides and other instruments for quantitative data collection were developed, translated and field-tested in Kabul district.

**Fig. 1:**
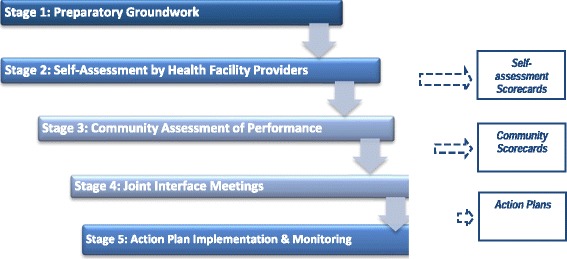
Fives stages of the community score card process

Figure 1 illustrates the five stages of the CSC implementation process. The *first stage; Preparatory Ground Work* includes building awareness amongst strategic stakeholders in the health facility and community, obtaining permission from local authorities and leaders and logistics planning. During this phase FGDs and key informant interviews were conducted in selected communities to describe the purpose and objectives of CSC and determine their interest in participating in the process.

In the *second stage; (Self Assessment by Service Providers)* facility health providers were invited to generate a self-assessment scorecard, prioritize indicators and subsequently assess their own performance. To ensure feasibility for improvement initiatives, they were instructed to prioritize 10–15 key performance indicators.

Subsequently, in *stage 3; Community Assessment*, participatory methods were used in both male and female FGDs with the nominal group technique to generate and prioritize 10–15 indicators for the CSC and eventually score performance on a scale of 0–10. The CSC facilitators solicited the support of the village leader, who ensured representative participation of male and female community members.

In s*tage 4; Joint Interface Meetings*, the results of the scorecards were shared by the community and health providers, with the other community representatives, with discussions on the rationale for the scores, followed by prioritizing indicators for the action plans. Indicators with the lowest scores and those considered important to both the community members and facility providers to ensure quality and utilization were prioritized. The research team facilitated the process to ensure equitable representation of ethnic minorities and women through community stratification and mapping, as well as selection of valid indicators based on BPHS standards. A ‘practice indicator’ was used to test the process. Scores were aggregated by the facilitator and recorded on a chart in the clinic.

The input or structural indicators included facility amenities, essential medicines, type of services, etc. This required a review of the BPHS standards, translation of relevant components, and educating the community about their entitlements and ‘patient rights’. The performance indicators included provider competency, courtesy and quality of care. During the interface meetings problems inhibiting the performance were openly discussed and recommendations and feedback collated and incorporated (Fig. 1). The CSC was an iterative process conducted every three months for scoring performance and monthly monitoring visits by the research team to resolve problems with the stakeholders. Three rounds of assessments were conducted in each facility and community.

In the final *Stage 5; Action Plan, Implementation and Monitoring*, providers and community representatives jointly determined an action plan and assigned responsibilities for improvements. Action plans were shared with the facility supervisor, provincial public health director, NGO manager, facility and community councils, and posted in the health facility. Activities described in the action plan were jointly monitored every month by the project team and relevant stakeholders in the community and facility. After a nine month process, qualitative evaluations, using FGDs were conducted in the selected communities to determine the value and perception of the CSC process and outcomes resulting from community engagement. These were coded and analyzed to generate the discussion themes. The research study was approved by the IRB in Kabul, MOPH and was considered exempt Human Subjects Research by the Johns Hopkins University IRB. Standard procedures for field surveys were followed to ensure confidentiality of participants, their opinions and safety procedures.

## Results

The study includes results from five facilities, as the team had to close one of the research sites to safeguard the survey teams in Takhar due to threats from the Taliban. As illustrated in Table 1, a diverse group of community members ranging from religious leaders, council members, school teachers, laborers, and housewives, engaged in the CSC process. During the initial stages, the facilitators sought participation by female community members, to ensure that their perspectives and opinions were considered. Male community members comprised 60 % of the participants in the study, but there was an increase in the proportion of female participants, between the first and third rounds in Takhar, (16.7 %), Sarqol, (20 %), Bakhtan, (25 %), and Kuzkunar, (16.7 %), and an overall increase of 43 % in Takhar for both males and females. To ensure effective participation, the number of CSC participants was limited to 6–12 community members, and with the harvest season during the third round, the numbers diminished in a few communities. On average, women scored physical amenities one to two points higher than their male counterparts. Male community members had higher expectations for the physical infrastructure of the facilities, except for availability of female doctors, who were deemed essential by both sexes. Provider courtesy, performance and quality received the highest scores.

**Table 1:** Scorecard participant profiles

CSC round	Community SC	n		Health provider SC	n		Interface meeting	n		Results	n	
		M	F		M	F		M	F		M	F
1	7	51	27	5	19	12	5	46	31	6	40	30
2	7	46	33	5	17	12	5	46	21	6	39	21
3	7	40	29	5	22	12	5	52	29	7	32	42
Total	21	137	89	15	58	36	15	144	81	19	111	93
Community scorecard participants	housewife, mosque mullah, salesman, school guard, student, teacher, vaccinator, village whitebeard, administrator, carpenter, council head, male council member, CHW head, malik, school manager, poet, farmer, health committee member, headmaster, Women’s council member, head of women’s council, driver, unemployed worker, veterinarian											
Provider scorecard participants	clinic supervisor, community health supervisor, laboratory technician, midwife, nurse, pharmacist, physiotherapist, vaccinator, doctor, counselor											
Interface/action plan participants	Clinic supervisor, CBHC officer, council head, headmaster, HMIS assistant, laboratory, midwife, nurse, pharmacist, physiotherapist, vaccinator housewife, student, teacher, malik, Imam, village whitebeard, women’s council director, farmer, carpenter community elder, architect											

*CSC* Community Scorecard, *SC* scorecard, *M* Male, *F* Female

At the onset, supervisors, providers, council members, and other leaders expressed considerable skepticism about the ability of community members, a majority of who were illiterate to identify indicators and score performance. Evidence of results in less than three months (i.e. following Round 1) convinced the majority of these ‘skeptics’ of the potential of the CSC process to address critical performance issues and enhance community ownership and responsibility for the facility, including the delivery of care.

Illustrative examples of CSC and action plans are demonstrated in Tables 2, 3 and 4 for three of the five facilities included in the study sample. Results from the other facilities appear in the Additional file 1. Scores assigned by the community members and providers for each indicator for the three rounds and the reasoning for the scores is also illustrated. Action plans likewise provide details about indicators prioritized by the team, persons responsible and a feasible timeline when it should be addressed, and notes on progress. In Siadara basic health facility, the major issues identified were the structural indicators on the physical condition of the clinic; damaged roof, water supply, and lack of electricity in the clinic (Table 2). Both providers and community members scored 5 in the first round as the water pump was damaged, once it was repaired, water availability received full scores. Similarly lack of power supply was scored low, and increased from 3 to 10, in the last round of scoring after the installation of solar panels. Five of the provider performance indicators received full scores (perceived provider competencies, punctuality, behavior, counseling, and waiting time). Following the interface meeting, where action plans were designed, the community, councils, providers and NGOs collaborated to facilitate the suggested improvements, demanding immediate action by the provincial directorate and governor. Aside from processing the requests to the community officials and authorities, provider- and community- led initiatives included the repairing of the facility’s roof, installation of solar panels for electricity and the establishment of a partnership with another NGO to assist in repairing the clinic’s water pump. Though some of the relatively simpler remedies, such as providing additional educational materials for patients in the clinic, were not addressed, providers and community members remained hopeful that they would be resolved in the near future. Initial scoring for quality of medicines was low, as the community was dissatisfied with the standard ‘white tablets’ prescribed to most patients, however, after being informed about the type of medicine and the BPHS standards their perceptions changed.

**Table 2:** Community scorecard and action plan, Siadara basic health facility, Bamyan

Indicators	CSC round			Reason		
	1	2	3			
Provider scorecard						
Broken water pump	5	10	10	Water pump repaired		
Non-use of ICE materials	8	10	9.5	Additional IEC materials and old copies replaced with new		
Damaged roof	9	8	10	Clinic’s metal roof repaired		
Medical equipment	7	6	7	Outdated medical equipment needs to be replaced		
Clinic cleanliness	7	9	9.5	Guard knows to prepare cleaning solution, but incinerator not available		
Waiting time	9	10	10	Low patient load; Separate outpatient department for males and females		
Clinic management	9	9.5	10	Function well as a team and the clinic is responsibly managed		
Accurate clinical exam	7	9	9.5	If patient volume is high then consultation time is inadequate, explaining poor quality for Antenatal care, Post natal care and IMCI		
Staff attitudes/behavior	10	10	10	No issues identified		
Community scorecard						
Water	5	10	10	Water pump repaired and safe water accessible to all		
Electricity	3	6	10	Initially, no electricity. PRT installed solar panels		
Medicines	7	10	10	Good quality, poor quantity. ‘White tablets’ given to all. Realized based on BPHS		
Staff competencies	10	10	10	Capable staff with good attendance record		
Waiting area	5	10	10	Patients forced to stand in the OPD until additional chairs were provided		
Staff punctuality	10	10	10	They are always present		
Staff behavior	10	10	10	Patients expressed satisfaction with staff interactions		
Patient counseling	10	10	10	Good medication management and counseling		
Waiting times	10	10	10	Reduced patient wait time		
Clinic cleanliness	10	10	10	Clean (i.e. no flies and garbage is disposed of regularly)		
Action plan						
Indicator	Action proposed			Who?	Time	Observations
Water supply	*Health council* request to NGO. Supervisor will coordinate with Yakawlang governor and local NGO			supervisor, NGO,shura PPHD	3m	Multiple negotiation meetings and follow up. Staff paid for pump from salary and eventually reimbursed
IEC materials	Supervisor requests staff to prepare a list of IEC materials and forward the request to NGO			supervisor NGO	1m	Follow-up requests, but materials not received
Physical condition, roof repair and electricity	Supervisor address issue with NGO and PPHD			supervisor, NGO, PPHD	1-6m	DHO and District Governor also engaged in processing the request. Community member assist in roof repair. Installation of solar panel for electricity with council and NGO
Equipment	Supervisor submits all departments’ requests for equipment needs to NGOs			Supervisor NGO	3m	Discovered chairs in storage and transferred to waiting area. Although *shura* not identified in AP, remained involved in all decisions
Clinic hygiene	Supervisor to train staff on infection prevention , create a plan for the clinic, and follow up			Supervisor, HF staff	Ongoing	NGO trained staff and assisted in developing action plan. Followed-up until 100% compliance
Medicines, exams and wait time	Patient triaging to avoid ‘noise’. Time spent with patient considered			HF staff	ongoing	BPHS: ≥ 9 min with patient. Guard engaged in patient triaging and guides patients to specific area

*DHO* District Health Officer PPHD Provincial Public Health Directorate Shura Facility council AP Action Plan

**Table 3:** Community scorecard and action plan, Sarqol basic health center, Bamyan

Indicators	CSC round			Reason		
	1	2	3			
Provider scorecard						
Clinic building	9	5	5	Request for clinic wall was not processed. Clinic was informed that the PPHD and NGO only had a budget for minor repairs.		
Ambulance	0	0	0	Expectation for all clinics to have an ambulance. Later learned that BPHS does not provide ambulances for BHC, but indicator remained in CSC.		
Equipment	9.5	5	4	Although usable, the delivery table is damaged. A request for a new one has not been processed. RHO and MOPH delegations are aware of the problem, but have failed to address it.		
Staff punctuality	10	10	10	Supervisor and midwife reside at clinic and are available at all times.		
Patient consultation	10	10	10	Appropriate care provided in each department.		
Patient wait time	10	10	10	Only patients with complex conditions wait an extended time period.		
Community scorecard						
Medicines	0	0	0	Previously, the medicines arrived late and community members were not aware of options. Now all medicines are available and effective.		
Ambulance	0	0	0	Urgent need for an ambulance, as clinic is located in a remote area. However, the BHC remains ineligible for an ambulance due to BPHS regulations.		
Clinic building	9	5	7	Clinic has no wall, but it is fairly large and now has more rooms.		
Patient beds	5	4	10	Beds available, but staff does not admit patients. Only one bed in delivery room. Additional beds were provided for patients and escorts.		
Laboratory	0	0	0	Clinics staff do not have access to laboratories, but this is not a requirement for BHCs under the BPHS guidelines.		
Waiting time	10	10	10	Patients do not wait and are examined in the order of arrival		
Patient counseling	10	10	10	Patients counseled in a sympathetic manner and provided treatment plans.		
Accurate exam	10	10	10	Patients are examined accurately.		
Staff punctuality	10	10	10	Staff arrives at clinic at 7:30 AM daily and serves the community throughout the night. Doctor and nurse are both present at all times.		
Clinic cleanliness	10	10	10	Support staff maintains a clean clinic. Sandals provided for patient use.		
Action plan						
Indicator	Action Proposed			Who?	Date	Observations
Availability of medicines	Staff raised community awareness of the types of medicines and ensured there were no stock outs			Staff, CHS, *shura*	…	Staff discussed BPHS guidelines and shared list of medicines with *shura* to ensure sufficient supply
Infrastructure, Clinic wall	Governor, PPHD and NGO, processing request.			PPHD	6 m	Insufficient resources to meet construction requests
Laboratory facilities	*Shura* request to supervisor Governor; PPHD and NGO process request.			Supervisor *shura*, NGO, PPHD	3 m	Mediation held with community and *shura* to illustrate that this was not required in guidelines
Replace old equipment	Supervisor submit request to NGO to replace delivery table and extra bed for escorts			supervisor midwife, NGO	1 m	Ongoing negotiations and new equipment promised for next year. Midwife/supervisor’s wife facilitated five more beds.

*RHO* Regional Health Officer

**Table 4:** Community scorecard and action plan, Kalafghan Comprehensive Health Center, Takhar

Indicators	CSC Round			Reason		
	1	2	3			
Provider scorecard						
Female doctor and nurse	9	8	4	No female doctor or nurse. Midwife overburdened, female patients cannot share many issues with male doctor. Increased to 8, as female nurse was hired, and decreased to 4 in last round as no female doctor was hired.		
Medicines	10	10	10	all medicines are available and arrive to the clinic on time. List expanded and efforts undertaken to make staff aware of essential medicines		
Waiting area	5	8	6	No suitable waiting area for males or females. A tent was provided by NGO staff, but winters are extremely cold and a metal roof is required		
Medical equipment	8.5	4	9	Inadequate and dysfunctional equipment (i.e. sphigmonometer, stethoscope, blood pressure cuff). In R3, the clinic received two BP monitors.		
Bathroom and guard room	0	1	3	Not selected in round 1. In round 2, location identified for the construction of bathroom for delivery room, round 3 construction initiated. A list of potential donors for the guardroom has been generated.		
Staff behavior	9	9	10	Patients satisfied with services provision. No difference between wealthy and poor. Staff behavior is good and they guide the patient to appropriate department		
Patient wait time	8	8	9	No suitable waiting area. Thus, not all can be counseled. CHC should be upgraded to DH. High patient volume with no female doctor and counseling takes more time.		
Education	9	9	9	Well-organized plan for each section. Proper prescribing. Still request more education.		
Staff Punctuality	9	9	10	Sometimes the staff do not attend clinic due to long distance and bad weather. In Round 2, all staff present during working hours and punctual.		
Community scorecard						
Clinic staff	9.5	8.5	7.5	Solved all problems. Should have received a 10. Understand the need for a female doctor after reviewing the BPHS.		
Waiting area	4.5	9	6.5	No proper waiting area - particular problem in summer and winter. Tent with few chairs.		
Medicines	4	6.5	9.5	Same white tablet for all patients. Now patients are more aware of the type and quality of medicines.		
Night staff	9.5	9.5	9.5	Solved all our problems when children were wounded. Staff is always available for treatment.		
Laboratory services	8	9	9.5	Laboratory technicians’ knowledge and skills were initially poor until they received the necessary training. As a result, laboratory tests have improved for urine, sputum and blood.		
Health education	9.5	9.5	10	Midwife is kind and provides advice during check-ups. On-time consultation provided to all and she speaks in simple language. Patients’ now have improved knowledge about health.		
Accurate exam	9.5	9.5	9.5	Patients are satisfied with the quality of care provided by the midwife. If she is not available, the doctor does not check accurately. Progress has been made and every patient is now examined appropriately according to illness.		
Staff punctuality	9.5	10	10	Patients reported overall satisfaction with staff performance, despite problems with absenteeism. Even on holidays and in the evenings, they are available and punctual.		
Action plan						
Indicator	Action Proposed			Who?	Date	Observations
Female doctors	*Shura* request that supervisor, NGO and PPHD directorate hire female doctor			supervisor, *shura*, CAF, PPHD	6 m	In a remote area, hiring of female doctor is difficult. Cultural barriers, such as rejection of non-Uzbek staff.
Waiting area	*Shura* request tent for male waiting area. *Shura*, supervisor and NGO staff request funds from community members via CBHC officer			supervisor, *shura*, NGO, PPHD	3 m	Community approached for donations. Promised to contribute, but not follow through. Budget remains insufficient.
Inadequate or old equipment	Supervisor request to replace BP monitors. CBHC officer report progress to supervisor and *shura*			supervisor, NGO, *shura*, HP staff	2 m	Initially MOPH did not allocate budget, now integrated in the budget. NGO provided 2 BP monitors
Guard- and bath- rooms constructed	*Shura* requests additional funds for construction from NGO through supervisor			supervisor, *shura*, NGO, CMs	6 m	Restroom constructed. *Shura* and supervisor received commitments from donors for guardroom.
Wait time decreased	*Shura/* supervisor request that NGO promote CHC to DH to hire a female doctor.			*shura*, NGO, CMs	6 m	Population is small; thus, upgrade request will be impossible to process
Accurate examination	supervisor request staff provide exams of sufficient duration and use appropriate equipment			HF staff	3 m	Due to staff shortages and no female physicians, this is not possible.
Staff availability	supervisor request night staff be monitored by NGO, CBHC officer and supervisor.			supervisor, NGO, *shura*	3 m	Staff satisfied with evening shifts.
Laboratory services	supervisor request training for laboratory technicians. Patient satisfaction monitored.			supervisor, NGO, *shura*	6 m	BPHC distributed job descriptions to community. Community satisfied.

In Sarqol, basic health center, both providers and community members requested that the clinic be upgraded to a comprehensive center and had higher expectations in terms of its infrastructure (e.g. purchasing of an ambulance, upgrades to laboratory facilities and the construction of a clinic wall). These indicators scored low, or zero in all rounds including the availability of ambulances and laboratory facilities (Table 3). Few of the infrastructure requests were fulfilled, primarily due to a lack of funding resources, including a compound wall, which was considered essential for the security of staff and patients. Interestingly the spouse of the clinic supervisor proved instrumental in securing additional beds for the delivery room, illustrating evidence of the widespread ownership of the CSC process and commitment to ensuring patient-centered care and improved quality. The scores for provider competencies and performance; (accurate exam, patient counseling, and staff punctuality), received a score of 10 in all rounds. The community members felt that the providers were conscientious and provided the best care for the patients considering the available resources.

The community members and facility providers prioritized the hiring of a female doctor at the Kalafgan comprehensive health center. Though a female nurse was hired, they were still dissatisfied. Improving clinic infrastructure was also a high priority. However, the patients expressed satisfaction with the accuracy of clinical exam (9.5 points) and appreciated the long distances providers travelled to reach the clinic, their punctuality and willingness to provide services at all hours of the night, especially the availability of night staff to offer services during emergencies. Although an NGO officer donated a tent to serve as a makeshift waiting area, separate male and female waiting rooms and adequate waiting areas remained a priority, as community members felt the tent would not suffice during the severe winter months, hence the score increased from 4.5 to 9, and decreased to 6.5 in the last round. Waiting time also remained a priority, but both staff and community members felt this could not be effectively addressed unless additional female doctors were hired for female consultations. Participants also requested increased training for laboratory technicians, as they were perceived to have limited competence.

The community health supervisors played a critical role in coordinating the meetings, including mailing letters. The participants remarked on how the CSC strategy was different from other programs and benefited the people. The level of participation of female community members surprised male providers and council members, as they seldom participated in a joint meeting with men historically. The availability of toilet facilities and other amenities, such as a laboratory, refrigerator, window screens, suction machines and a water tank were prioritized in Bakhtan and Kuz Kunar in Nangarhar province. Access to a physiotherapist was also voiced in Kuz Kunar. The supervisor and NGO eventually facilitated visits from a physiotherapist, twice a month.

Following the completion of the CSC phase, qualitative evaluations were conducted in these communities using FGDs to determine the value and outcomes of engaging in CSC. The findings illustrated positive outcomes in terms of equitable community participation, opportunities to share their opinions, the ability to jointly identify and address problems through the development of action plans; and improved service utilization, particularly facility deliveries (Table 5).

**Table 5:** Selected illustrations from focus group discussions on CSC strategy

Theme 1: Engagement in the CSC process
Sub-theme: Gender and equity in CSC participation
Codes: Influence of family support (husbands and mothers-in-law); socio economic equity (participation of members of all socioeconomic strata); Equitable participation/involvement of women; Equitable participation/involvement of all members of other minority groups (i.e. religion, ethnicity, etc.) (excludes women); Reiteration of gender roles; Women’s subordination
Illustrative Quotations:
We have no discrimination. All of our people, they are poor or rich, are participating in the meetings according to the need for their participation, specially the whitebeards who have more experience of life.
- Community member (female), Zir Shakh village
Both poor and rich people participated…and eat from one plate. - Community member (male), Norka village
In the first round, women participated more than men. But, in the 2nd and 3rd, men and women were equally represented and participated – Health council member (male), Norka village
All of them were participating, even women were more in number than men in some meetings.
– Community member (male), Minar Sofla & Olya village
Currently you see the head of development council and teacher and also some others are present in this meeting and they participating actively in other meetings too. – Community member (male), Zir Shakh village
As you witnessed, elders and influential community members all participated in the meeting.
- Heath post council member (male), Norka village
Community elders, including the head of the community development council and a white beard, were present at that meeting, and they actively took part in the gathering. – Health clinic staff (female), Zir Shakh village
Men and women both were coming to the meetings. The head master of the girls’ school always used to participate in the meetings and women were more active regarding participation in the meetings, than men.
- Health facility council member (male), Sarqol village
Sub-theme 1.2: Barriers to CSC participation
Codes: Distance to clinic; Availability of transport; Security; Weather; Scheduling of FGDs (dates and times); Awareness of CSC; Influence of others on participation (e.g. mother-in-law, husbands)
Illustrative Quotations:
Some people who are living in far places from the clinic or are employed; it is difficult to them to participate in these meetings. – Community member
Now our clinic is improved. When the community health supervisor informed us about the meetings or any other matters related to the clinic, we would leave our urgent work to come and participate. The main problem is the far distance to get to the clinic. –Community member (male), Norka village
It took three hours for me to reach the clinic by foot. People have problems of long distance and cold weather. –Health facility council member (male), Sarqol village
There is long distance and it is winter season so the people who are living near here can participate in the meetings. - Health facility council member (male), Sarqol village
Sub-theme 1.3: Awareness of the types of health services and entitlements under BPHS
Codes: Knowledge of the roles and responsibilities of service providers; Understanding of entitlements guaranteed under the government services; Health education/literacy; Gaps in service coverage
Illustrative Quotations:
When we participated in the meetings, we were aware of the service hours for the providers, previously we thought they only worked till lunchtime. – Community member (female), Zir Shakh village
Previously we were not aware about the monthly expenditures of the clinic for generator fuel, CHW monthly meetings, transportation costs, and clinic repairs, after participating in the CSC, we are aware that the NGO provides 9000 Afs to the clinic for these expenditures and it should be spent in consultation with the Shura for more transparency - Health council member (male), Sarqol village
Theme 2: Perspectives of healthcare services and health providers (i.e. health care experience)
Sub-theme 2.1: Perceptions of health services and quality of care
Codes: Health facility staffing; availability of female providers; Infrastructure; Provider behavior and competency to provide quality care; Preferred (first) source of care; Equipment and supplies; Disparities in care (sex, socioeconomic status); Waiting time; Availability of specialists ; Effectiveness of medicines and treatment; Expectations for care; Satisfaction with care; Increased use of facility by members of rural/remote communities; Increased use of facility by pregnant women; Facility as source of pregnancy-related information and/or education, including the importance of facility-delivery
Illustrative Quotations:
As the information about the clinic’s services is given to the people, nowadays people are increasingly going to the clinic and even pregnant women give birth in the clinic. – Community member (female), Zir Shakh village
Nowadays more people are coming to the clinic and receive medicines and they are not going to other places for treatment. – Community member (male), Minar Sofla & Olya village
All people are coming for medicines to the clinic and especially pregnant women are coming to seek advice and giving birth in the clinic. – Community member (female), Minar Sofla & Olya village
More patients are now coming to the clinic and people even from remote areas, such as Lal and Ghor, come to this clinic for treatment. – Health council member (male), Norka village
The quality of health services improved after your meetings…It has been said that pregnant women now go to the clinic for giving birth. – Health council member (male), Sarqol village
It is the first time we learnt about safe delivery and the importance of delivering with a midwife in the clinic and not at home – Community member (female)
Sub-theme 2.2. Perceived trust in providers and decision making
Codes: Decision-making processes; Transparency; Communication; Support; Respect for patients’ rights/privacy; Trust in facility staff; Acceptability of health information provided; Accuracy of health information provided
Illustrative Quotations:
We trust the clinic personnel and clinic personnel are giving accurate information.
– Community member (male), Norka village
After providing water and electricity for the clinic as well as good behavior of clinic personnel, people trust the CHWs and clinic personnel more. - Health council member (female), Norka village
Transparency, understanding and trust have been created. The affairs which have been done or not, we discuss and resolve the problems. - Health council member (male), Chinar-e-Gungishkan village
Theme 3: Perspectives on CSC Effectiveness and Action Plans
Sub-theme 3.1. Perceived effectiveness of the CSC Strategy
Codes: Health care utilization; Quality of care;. Follow-up monitoring; Tracking progress; Use of data to inform decisions (i.e. maintaining and utilizing records); Appreciation for health performance metrics;. Increased and strengthened relationships between the community and providers
Illustrative Quotations:
We learned more, we understood what an indicator is, what is input, what is performance, it’s the first time we learnt such terms. - Community member (male), 50 years old and employed as a farmer
The CSC is a good program, like a bridge between the community and clinic. We (community) were on one side of the river, and the clinic was on the other side, the CSC is like a bridge that connected us.
- Community member (male), shopkeeper
It was very good practice that they were writing it on the paper and we could find solutions for the problems together with clinic personnel. – Male health council member, Chinar-e-Gungishkan village
Sub-theme 3.2. Ownership of and Accountability to the CSC process
Codes: Accountability (positive and negative) among health care providers and community; Solidarity and shared governance, responsibility/ownership of facility
Illustrative Quotations:
The sense of ownership has increased. According to the sayings of Mr. Bihishti people got lazy and always waiting for NGOs to come and dig some well for them, or construct road and concrete stream for them. People should wake up and actively participate in any activity. - Community member (female), Minar Sofla & Olya village
Before some people used to utilize the building for wedding ceremonies and a power broker used to park his vehicle in the clinic. Till the CSC meetings, we didn’t understand that the clinic is our property and we are responsible for its protection. - Community member (female)
Now people perceive the clinic as their own property and are trying their best to complete the clinic’s surrounding walls… - Community member (male), Minar Sofla & Olya village
People now take care of the clinic even better than their homes. – Health council member (female), Norka village
People know that the clinic belongs to them and should be well kept. –Health council member (male), Norka village
We feel more and more the ownership of our clinic. Clinic is like our own house and each community member should ask about the services of the clinic.
– Health post council member (male), Chinar-e-Gungishkan village
It is true that people now perceive that the clinic is their own property. When mothers are going alongside with their children inside the clinic, they not allow their children to touch the clinic walls and the clinic building’s glass, to not be scratched or broken. – Health post council member (male), Shura, Sarqol village
After the three rounds, people trusted the clinic increasingly. Meetings also created a sense of ownership of the clinic. They say that before they thought that the clinic belonged to the government and they were not aware of its affairs. Now they are aware about all clinic issues and they know that the clinic belongs to them.
– Health facility staff (physician), Kalafgan
Our clinic building does not meet standards or have a waiting place. Thanks to the CBHC officer who brought a tent, our problems lessened…during the spring and summer. In the winter, it is impossible to use, because it is far too cold. – Health facility staff (supervisor), Kalafgan
Sub-theme 3.3. Added value of the CSC process
Codes: Improvements in the provision and delivery of care; Strengthened community-provider relationships;. Increased availability and accessibility of care; Enhanced gender engagement; Governance and transparency
Illustrative Quotations:
This program is excellent. It gives women a forum to share their experience and perspective on healthcare. If we work with them and inform them, it’s better for the future of the health system. In Bamyan, we had joint male and female meetings. We are a closed society. Of the many projects I have worked on, this is the first time I witnessed this. – CBHC team member
Before this program, vacancy announcements for a female doctor were only on the signboards of the NGO’s office and the PPHD, but now they are in other places, such as hospitals and clinics. – Health facility staff (physician), Kalafgan
This is the best program. Through it, people get information about all programs, personnel and services of their related health facilities. For example, people were not aware about clinic affairs. Now they know many doctors a clinic should have and what services it should provide. – Health facility council member
Theme 4: Opinions of the sustainability and scale up of the CSC strategy in other communities and regions
Sub-theme 4.1. Sustainability of the CSC approach
Codes: Factors contributing to the CSC sustainability ;. Benefits of sustaining the CSC approach;. Stakeholders critical to CSC sustainability; Factors contributing to the CSC scale up; Benefits of CSC scale-up
Illustrative Quotations:
This program should be developed across all of Afghanistan. In the past there was measles disease in our area. Now that we have clinics, children are vaccinated and measles are eliminated. It is very good to extend the program for all provinces of Afghanistan whether it is south or east; in Kandahar and Herat as they are all our brothers. – Community member (male), Norka village
If people participate in the meetings, solidarity will be stronger. – Community member (female), Zir Shakh village
The program should be expanded to all provinces of Afghanistan and this program should run countrywide in Afghanistan. – Health council member (female), Chinar-e-Gungishkan village
This program should continue in all Afghanistan. All afghans are our brothers and their health is important like our health. Each resident of Afghanistan should get benefits of this program.
– Health council member (male), Sarqol village
[From a community perspective], this is a really good project even better than PDQ. It is also different from FFSDP and BSC, because your indicators are selected by the community and providers. There was client satisfaction within BSC, but not like CSC. – NGO staff

### Gender and equity in CSC participation

*“Men and women both were coming to the meetings. The head master of the girls’ school always used to participate in the meetings and women were more active regarding participation in the meetings, than men”*

*“We have no discrimination. All of our people, they are poor or rich, are participating in the meetings according to the need for their participation, specially the whitebeards who have more experience of life”*

### Linking communities with health facilities

*“the CSC is a good program, like a bridge between the community and clinic. We (community) were on one side of the river, and the clinic was on the other side, the CSC is like a bridge that connected us”*

*“before some people used to utilize the building for wedding ceremonies and a power broker used to park his vehicle in the clinic. Till the CSC meetings, we didn’t understand that the clinic is our property and we are responsible for its protection”*

*“Transparency, understanding and trust have been created. The affairs which have been done or not, we discuss and resolve the problems”*

Overall, participants voiced that the utilization improved, resulting from increased ownership and accountability among all individuals engaged in the process. Many respondents stated that the clinic was now their ‘home’. Though many of the participants unanimously agreed in scaling up this strategy in the rest of the country, their primary concern was the long distances they had to travel to participate in the interface meetings, and suggestions were made to hold these meetings in the mosques or schools in the community. The lack of financial resources for addressing infrastructure deficiencies was mentioned as a limitation.

Despite some initial concerns about the exclusion of villages in remote locations, the community, providers and council members commented that even the most marginalized groups had access to and participated in the CSC process. Participants also remarked on how the CSC was different from other projects, including the National Solidarity Program, as it enhanced participation by community representatives. The NGO involved in the study played an important supportive role and accommodated the survey teams and facilitated the logistics. The facility councils were instrumental in mobilizing the community members for the interface meetings, and though not mandated, were present in every interface meeting.

Considering the remote and insecure location of the selected communities, the responses indicated a sense of community solidarity, ownership and trust and several remarked that it was the first time they have been involved from inception to execution of a project as opposed to just participating in evaluations and that it was conducted in a transparent manner. The constructive dialogues, helped communities appreciate the challenges faced by providers in delivering quality care and likewise the providers recognize the needs and expectations of patients.

## Discussion

Results from the CSC study provide some promising evidence of the relevance and importance of engaging communities for social accountability to enhance equitable access, quality and coverage for healthcare, and offer support to the management oversight of health facility operations especially in fragile contexts. This has important implications especially for Afghanistan, as development is hampered by a myriad of factors including an unstable political system, poor economy, and ongoing violence, which negatively influence health care provision [21]. Despite the impressive gains achieved over the past decade, the future of Afghanistan’s healthcare remains at risk, as the country has been dependent on external assistance in the past and diminishing donor aid may reverse the achievements in coverage and quality. Rural communities have poor access to formal health services, and the Ministry and NGO’s have attempted to populate the villages with an extensive cadre of community based providers for basic preventive and curative care, aside from the establishment of sub-centers and mobile health care services. The chronic deficits in professional health workforce, particularly the demand for female providers (<25 % of the workforce) poses additional constraints for optimal healthcare delivery [22].

Case studies from other low- and middle- income economies have demonstrated success in healthcare access and service quality, when communities have been involved in the generation of performance scorecards and participated in the management oversight of clinics [23]. The present study, has also demonstrated similar potential, for fragile contexts like Afghanistan, facing acute shortages in capacity and service provision. The CSC process enables active community participation in generation of key performance indicators, voicing community and health provider concerns, public disclosure of information to improve transparency and accountability and joint decision making to enable improvements. The dissemination and advocacy of the action plans with senior authorities provides an opportunity to assess factors influencing performance and feasibility of appropriate remedial measures and the restructuring of policies and implementation procedures.

Civil societies in most low- and middle-income countries are becoming more engaged in advocacy and government accountability. In some countries, donor aid is being channeled through national governments rather than through NGOs, necessitating additional mechanisms of accountability and transparency. With the withdrawal or diminished external military forces, donors are anticipating options for government autonomy in managing donor resources, requiring more active participation of civic engagement. The CSC, if executed through the ongoing mechanisms of community based healthcare, can prove to be a valuable tool, toward instituting accountability measures for health system performance as it facilitates the flow of information between citizens and service providers, as evidenced in Vietnam and other African countries [16, 17, 19]. As an instrument of social and public accountability, implementers need to consider the social and political context, ensure skilled facilitation, and awareness of opportunities for participation among all ethnic and socioeconomic minorities prior to the launch of the CSC [24]. The close and frequent mentoring of trained facilitators is a critical prerequisite for ensuring the gains in this process, and have to be conducted by those that are not officially affiliated with the government, but NGO’s and other academic or civil society organizations.

The CSC process resulted in the participation of stakeholders and all levels of the health systems and generated a greater sense of community solidarity and partnership. The interface meetings served as a key strategy for community engagement, inspiring a sense of self-help among participants, which resulted in both non-monetary and financial contributions for improvements. Aside from addressing community grievances about capacity and quality of services, the process created awareness of BPHS through a ‘rights based approach’ to empowerment [25].

The CSC process promoted social accountability through the participation of community members and leaders, making them responsible for their health and created an enabling environment to share opinions, perspectives and recommendations and engage in a non-threatening manner to jointly develop action plans for local policy. The participatory nature of the CSC process in determining input and performance indicators and scoring built skills and competencies for community based performance monitoring. The multi-stakeholder engagement of facility supervisors and providers, male and female community members, facility and health post community councils, community leaders, NGO managers, CBHC coordinators, provincial directorates in the execution of the action plan promoted community solidarity and shared vision to enhance the quality of service delivery.

At the community level, the most critical contribution was the increased awareness of rights guaranteed under the BPHS. Facility amenities, such as functional and hygienic restrooms, gender segregated waiting areas, running water, a stable power supply and security measures (i.e. a compound wall), were important factors for accessing quality healthcare. Interestingly, facility cleanliness was a high priority indicator for most communities. Voluntary contributions by individuals and initiatives by committees to undertake the repairs were outstanding features of the CSC process. The ‘self-help’ attitude and ‘self-realization’ to promote change in the delivery and overall quality of care and that they are able to make a difference was not previously evidenced in these extremely marginalized and vulnerable communities.

**Fig. 2:**
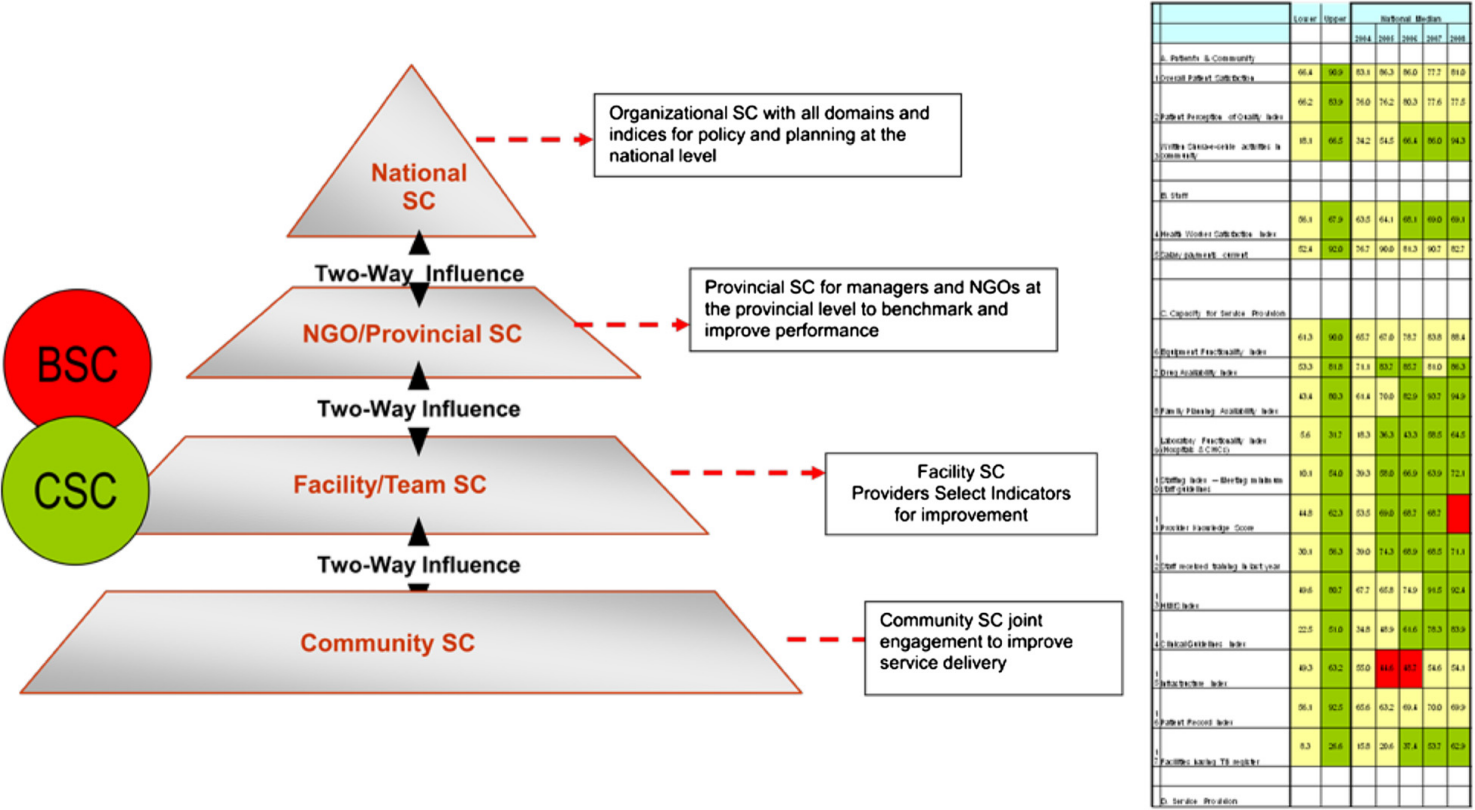
Cascading performance scorecards across the health system pyramid

Results from this study indicate many positive returns, especially increased engagement of female community members and enhanced sense of mutual trust between the community and providers. Culturally appropriate community-level monitoring tools like the CSC enable low literacy communities to determine patient centered measures of quality and care, score performance, and institute remedial action at the facility level [19]. The process of jointly identifying priorities and actions empowered patients to take ownership of their own health and the way in which they access care, while motivating providers to make changes to the way in which they deliver care and advocate for changes to the facilities in which they operate [25]. This experience was also evidenced by similar projects implemented by the World Bank in the Philippines, where the ‘bottom-up’ assessment of services motivated providers to improve services [26]. Another critical factor that emerged from the CSC process is the ability to interpret quality in an ‘everyday’ language with indicators that are easier to comprehend, than the traditional indicators of provider patient relationships [27]. Successful integration of the CSC can complement the ongoing BSC at the facility level and cascade performance strategies through the health system to meet the objective of making strategy everyone’s everyday job, as envisioned by the innovators of the BSC [Fig. 2].

Five critical elements for successful execution of the CSC were identified.

### Aligning other ongoing priorities of the Ministry of Public Health and the NGOs

The healthcare landscape in Afghanistan is undergoing dynamic changes in policy and service delivery hence the CSC needs to be aligned with other ongoing strategies. NGO’s are under pressure to innovate cost effective mechanisms to achieve the goals and performance targets. Consideration must also be made for the indicators targeted by the health management information system, which may be different from those prioritized by the CSC.

### Capacity for skilled facilitation

Another important element is the participatory process, which integrates feedback, dissemination and follow-up by all concerned stakeholders and adaptation to the local context. The technical capacity of the organization adapting the process must be ensured, including the availability of skilled facilitation and experience with participatory approaches. Facilitation must include the ability to mobilize and ensure buy-in from the providers, communities and their leaders as well as local authorities.

### Considerations for insecure and remotely located communities

With the increasing threat of attacks on health facilities and providers, security concerns are a major impediment, and leaders and healthcare providers must be vigilant in ensuring that community members are not at higher risk for participating in collaborative activities with the health clinics.

### Generalizability of the CSC process

Selection of community participants must have some element of randomness and representativeness. The initial FGDs to generate the CSC indicators must be conducted in a neutral and safe environment, where participants can freely discuss their opinions. The interface meetings require skilled facilitation especially during the launch of the CSC and the initial rounds to balance the expectations and opinions of service providers and community members without compromising the ‘voice’ and transparency of the process. For future sustainability, the roles could be assumed by facility councils who are increasingly involved in providing management oversight and governance to facility operations. Significant differences were found in quality of pediatric care in facilities reporting functional health councils controlling for other factors [28].

### Addressing CSC limitations

Skilled facilitation is essential to balance community demands and expectations with the capacity and ability of the local health system to generate improvements. The requests for ambulance, female doctors, enhanced diagnostic capabilities may be legitimate, yet unattainable, goals. A few providers raised concerns that enhanced utilization would increase patient load and they would be unable to cope with the demands. This requires appropriate triaging and referral systems to optimize the CHW services. If the CSC is accepted as one of the national strategies for community engagement in national policy, the initial training and facilitation will be provided by the existing mechanisms through provincial CBHC officers, the Community health supervisor and more strategic engagement of the facility councils. A few local NGO’s have expressed interest in training to serve as facilitators for future execution of the CSC. These measures would minimize some of the transport costs. However, a minimal cost of approximately $300–$500, per facility will be required annually, for stationary and other supplies required for the interface and action plan meetings.

Access to facilities was quoted as a main impediment to participation as community members in some areas had to walk over three hours to attend the meetings, and suggestions were made for mosque based meetings, especially to ensure that the ‘voice’ and opinion of women are considered.

Dissemination of the results with some of the policy makers generated considerable interest on the potential of the scorecard, though there was some skepticism about the generalizability due to the small sample and ethnic diversity of communities in Afghanistan. The CBHC Department is considering institutionalizing the CSC strategy as one of the government’s recommended strategies for community engagement. Enhancing community participation and accountability through these complementary mechanisms can promote positive heath practices and motivate the most marginalized and vulnerable citizens to access health services, better advocate for their needs, and reinforce accountability [19, 27, 29]. Like other countries in post-conflict, weak health systems, which are characterized by extreme resource deficiencies, rely on the governance of local authorities and health councils, and hence the CSC needs to be locally adapted to ensure sustainability and legitimacy [30]. As performance based financing initiatives are being scaled-up in some provinces, the CSC holds promise as a health system governance and accountability tool.

## Conclusion

Like other developing countries, Afghanistan faces a myriad of structural, social and economic challenges which impede the achievement of universal coverage for healthcare. The preliminary findings indicate that the CSC process provides a complementary strategy for the integration of community and patient perspectives in the care process, by instituting culturally appropriate governance mechanisms to engage providers, community and other stakeholders to enhance the performance of the local health system. The contextualization of the CSC process and results is a critical factor for scale up, as illustrated in the principles of implementation science, and will require further consideration and empirical research for policy integration [31, 32], [33].

## Additional file

Additional file 1: Table S1. Community Scorecard and Action Plan, Bakhtan Basic Health Center, Nangarhar province. **Table S2.** Community Scorecard and Action Plan: Kuz Kunar Comprehensive Health Center, Nangarhar province. (DOCX 24 kb)

## Abbreviations

CBHC: Community Based Health Care Department
CHW: Community health workers
BPHS: Basic Package of Health Services
CSC: Community scorecard
BSC: Balanced scorecard
MOPH: Ministry of Public Health
NGO: Non Governmental Organization
