# Supplementary material for: Enhancing governance and health system accountability for people centered healthcare: an exploratory study of community scorecards in Afghanistan
Source: BMC Health Serv Res. 2015 Jul 31;15:299. doi: 10.1186/s12913-015-0946-5 (PMC4521484; doi:10.1186/s12913-015-0946-5)
Supplement: Additional file 1: Table S1. — Community Scorecard and Action Plan, Bakhtan Basic Health Center, Nangarhar province. Table S2. Community Scorecard and Action Plan: Kuz Kunar Comprehensive Health Center, Nangarhar province. (DOCX 24 kb) [file 12913_2015_946_MOESM1_ESM.docx]

Enhancing Governance and Health System Accountability for People Centered Healthcare: An Exploratory Study of Community Scorecards in Afghanistan

BMC Supplemental Files

Table S1: Community Scorecard and Action Plan, Bakhtan Basic Health Center, Nangarhar province

| *Indicators* | *CSC Round* | | | | | | *Explanation* | |
| --- | --- | --- | --- | --- | --- | --- | --- | --- |
|  | ***1*** | | ***2*** | | ***3*** | |  |  |
| ***Provider scorecard*** | | | | | | | | |
| *Damaged water tank* | | 4 | 10 | | 10 | | | Water tank repaired |
| *Waiting area* | | 5 | 8 | | 10 | | | Waiting area constructed, equipped with concrete seats |
| *No public bathroom* | | 2 | 8 | | 10 | | | Toilet constructed, necessary tools brought by HN-TPO |
| *Window screens* | | 1 | 7 | | 10 | | | Screens installed on clinic windows |
| *Suction machine broken* | | 9 | 10 | | 10 | | | Suction machine repaired by staff of the clinic |
| *Accurate exam* | | 7 | 10 | | 10 | | | Number of health education sessions increased with help from *Shura-e-Sehi* and community members. Accurate exams are being conducted |
| *Community health supervisor fuel, top up card* | | 3 | 3 | | 3 | | | Request made to HN-TPO, as items were not part of policy. Offered to provide bicycles instead. Not practical solution. Bakhtan BHC reported that its request for a bicycle was approved. |
| *CHW referral forms* | | 7 | 10 | | 10 | | | Neglected. Greater attention paid to the referral sheets |
| ***Community scorecard*** | | | | | | | | |
| *Waiting area* | | *4* | *9* | 10 | | Waiting area constructed, equipped with concrete seats | | |
| *Electricity* | | *5* | *7* | 9 | | Directorate of electricity in Nangarhar invited elders to sign an MOU for electricity | | |
| *No toilet for patients* | | 0 | *5* | 10 | | Toilet constructed, necessary tools brought by HN-TPO | | |
| *Clinic staff punctuality* | | *7* | 10 | 10 | | *Clinical staff is now punctual* | | |
| *Behavior with patients* | | *9* | 10 | 10 | | *Attitudes/behaviors towards patients improved. No complaints made.* | | |
| **ACTION PLAN** | | | | | | | | |

| Indicator | Action Proposed | Who? | Date | Observation |
| --- | --- | --- | --- | --- |
| Waiting area | Shura to request during 1st meeting and send approval to NGO responsible for BPHS in Nangarhar | CIC, *Shura*, HN-TPO, PPHD | 2m | Completed |
| Public toilet | *Shura* approve request and select a place for construction in collaboration with community | CIC, *Shura*, HN-TPO, PPHD | 2.5m | Completed |
| Water tank repaired | CIC to address it with NGO and PPHD | CIC, *Shura*, HN-TPO, PPHD | 20 days | Completed |
| No window screens | CIC should request nets from *Shura* and send approval to HN-TPO for action | CIC, *Shura*, HN-TPO, PPHD | 1.5m | Completed |
| Accurate exam | Facility staff should include issue in weekly meeting agenda and follow through accordingly | CIC, *Shura*, HN-TPO, PPHD | 2.5m | Completed |
| Community health supervisor fuel, top up card | To be included on clinic’s agenda during next meeting and information sent to the HN-TPO | CIC, HN-TPO | 2.5m | Incomplete |
| CHW referrals | *Shura* to raise the issue during their meeting and facility staff consider it during their daily performance | HC staff, *Shura*, CHWs | 2.5m | Completed |
| Staff attitudes towards patients | *Shura* to raise the issue during meetings and follow-up with facility staff | HC staff, *Shura*, HN-TPO | 2.5m | Completed |
| Staff punctuality | CIC to discuss issue during the weekly meeting in presence of HN-TPO | CIC | 2.5m | Completed |

Table S2: Community Scorecard and Action Plan: Kuz Kunar Comprehensive Health Center, Nangarhar province

| *Indicators* | *CSC Round* | | | | *Explanation* | |
| --- | --- | --- | --- | --- | --- | --- |
|  | ***1*** | ***2*** | | ***3*** |  |  |
| ***Provider scorecard*** | | | | | | |
| *No window screens* | 9 | 10 | | 10 | | Nets were ultimately installed and have spare nets for future use |
| *Laboratory refrigerator* | 0 | 5 | | 8 | | No major incidents of blood transfusion in the clinic. Have an ambulance for referral if necessary. |
| *Imbalanced salaries* | 9 | 9 | | 10 | | Committed to national salary policy of the Ministry. Request that HN-TPO revise the contracts so that the salaries align |
| *Clinic hygiene* | 9 | 10 | | 10 | | Attention paid to hygiene and windows cleaned |
| ***Community scorecard*** | | | | | | |
| *Availability of medicines* | 4 | 5 | 5 | | | *Inadequate and patients and providers dissatisfied with the availability* |
| *Waiting area* | 5 | 6 | 8 | | | *Temporary solution – clinic staff still seeking permanent solution* |
| *Ltd access to therapist* | 5 | 5 | 8 | | | *Now comes to clinic twice a month* |
| *Ltd night duty staff* | 5 | 5 | 7 | | | Clinic staff providing health education to communities requesting that women in labor be brought to clinic in the evening hours |
| *Patient exam* | 8 | 7 | 10 | | | Adequate clinic examination |
| *Staff attitude* | 6 | 4 | 9.5 | | | Males are satisfied. Females believe that they have to provide them with complete medicine and behave more professionally. |
| *High patient volume* | 4 | 4 | 6 | | | Will send request to PPHD through *shura-e-sehi* to upgrade to district hospital |
| *Staffing levels* | 10 | 10 | 10 | | | Fully staffed and punctual |
| *Medical equipment* | 10 | 10 | 10 | | | Complete equipment |
| **ACTION PLAN** | | | | | | |

| Indicator | Action Proposed | Who? | Date | Observation |
| --- | --- | --- | --- | --- |
| Waiting area | Separate male and female waiting areas for patients developed in coordination with CIC, CHS and *shura* | CIC, *shura*, CHS | 2.5 m | Completed |
| Clinic hygiene | Hygiene discussed at weekly staff meetings and progress followed-up | HF staff | 1.5m | Following net installation, hygiene improved, but issues arose recently. |
| Availability of medicines | Clinic staff should request adequate medication based on HMIS reports | CIC, pharm tech | 2.5m | Completed |
| Window screens | CIC should identify and request nets from HN-TPO | CIC, HN-TPO | 15 days | Completed |
| Physiotherapist available | To be addressed via the *shura* and approved by CIC and sent to HN-TPO for further action | CIC, *shura*, HN-TPO | 2.5m | Completed |
| Night duty | Raised by CIC during weekly meetings, emphasizing need for a schedule based on requests of the HN-TPO and *shura* | CIC, HN-TPO, *shura* | 2.5m | Completed |
| Patient volume | Requests to upgrade to district hospital should be made via the *shura* and district governor office | CIC, *shura*, district governor | 2.5m | At interface, promised to invite *Shura* and request upgrade |
| Perception of staff attitudes | CIC should monitor patient flow to assess provider behavior | Clinic staff, *shura*, HN-TPO | 2.5m | Completed |
